# Detection of Epigenetic Variations in the Protoplast-Derived Germlings of *Ulva reticulata* Using Methylation Sensitive Amplification Polymorphism (MSAP)

**DOI:** 10.1007/s10126-012-9434-7

**Published:** 2012-02-10

**Authors:** Vishal Gupta, A. J. Bijo, Manoj Kumar, C. R. K. Reddy, Bhavanath Jha

**Affiliations:** Discipline of Marine Biotechnology and Ecology, CSIR—Central Salt and Marine Chemicals Research Institute, Bhavnagar, 364002 India

**Keywords:** Epigenetic variation, DNA methylation, Protoplast, MSAP, *Ulva*

## Abstract

Regeneration of protoplasts into de novo plants was reported for a large number of seaweed species. The regeneration of protoplasts into different morphotypes as a result of epigenetic variations was discussed for the first time in this study. The loci assessed for methylation modifications in normal filamentous thalli showed a frequency of 32.43% as unmethylated DNA, 24.32% as a hemimethylated, and 20.27% as a methylation of internal cytosine at both the strands. The corresponding methylation values for disk-type thalli were 27.02%, 32.43%, and 14.86%, respectively. The hypermethylation condition was apparent in the disk-type thalli with methylation ratio of 72.97% compared to that of normal filamentous thalli with 67.56%. The frequency of methylation polymorphic sites among the two morphotypes was 53%. The present study reveals the distinct expression of cytosine methylation and is thus correlated to differential morphogenesis of plants regenerated from cultured cells. The number of protoplasts regenerating into filamentous thalli declined with increasing temperature from 15°C, 20°C, 25°C, and 30°C. The disk-type variant had higher thermal stability at 30°C over normal filamentous thalli. Further, this variant could maintain itself for more than a year in the laboratory indicating its suitability for in vitro germplasm maintenance and propagation.

**Electronic supplementary material:**

The online version of this article (doi:10.1007/s10126-012-9434-7) contains supplementary material, which is available to authorized users.

## Introduction

Plant protoplasts have been employed for investigating the various aspects of developmental biology and in vitro genetic manipulation techniques aimed at development of genetically improved strains of agronomic crops. There have been numerous studies on the isolation and regeneration of protoplasts from a wide variety of seaweeds ranging from morphologically simple leafy thallus to anatomically complex thallus (see review Reddy et al. 2008). Unlike higher plants, seaweed protoplasts regenerate and differentiate into a full thallus without any amendments of phytohormones to culture medium. Nevertheless, the protoplasts from green seaweeds followed different types of regeneration patterns and gave rise to several phenotypically variable morphotypes such as sporangia, microthalli, saccate (or spherical), tubular (or spindle), irregular, and frondose with various life spans (Reddy et al. 1989; Huang et al. 1996; Chen 1998; Krishnakumar et al. 1999; Chen and Shih 2000; Rusig and Cosson 2001). Also, in the red alga *Porphyra*, three types of protoplast regeneration patterns, i.e., callus, filamentous, and conchocelis have been described (Polne-Fuller and Gibor 1984; Fujita and Migita 1985; Waaland et al. 1990; Dipakkore et al. 2005). The reasons offered for differentiation of cells into such variable morphotypes in vitro were mostly speculative and primarily attributed to either axenic culture conditions employed (Singh et al. 2011), age of source material used, or physical culture conditions such as temperature and irradiance (Chen 1998).

Although in vitro conditions do induce phenotypic variations, it is not well understood how various morphotypes develop from a single genotype when cultured under the same conditions (Vogel 2005). Studies on higher plants have shown that the epigenetic mechanisms such as DNA methylation, histone modifications, chromatin remodeling, and RNA interference regulate the differentiation and development of cells or tissues cultured in vitro. These epigenetic mechanisms influence the expression of genes that in turn triggers the signals involved in development program eventually leading to formation of different phenotypes. It has also been suggested that in vitro conditions induce genotypic variations at modest frequency while variation in degree of DNA methylation seems to be frequent and occasionally directly linked with phenotypic variations (Miguel and Marum 2011). Studies on terrestrial plants have revealed that epigenetic regulation controlled by frequency and distribution of DNA methylation causing pleiotropic effect on morphology and development (Zhang et al. 2006). The DNA methylation mostly occurs at cytosine particularly at CG dinucleotides, although significant levels of methylation at CNG and CNN has also been reported (Cao and Jacobsen 2002; Hsieh and Fischer 2005). Differential DNA methylation regulates the specific gene expression by getting associated with the 5′ upstream promoter sequence either in one or both alleles of tissue-specific genes (Fraga et al. 2002). Significant differences in cytosine methylation have been observed in different parts of a species such as tomato (Messeguer et al. 1991), rice (Xiong et al. 1999), *Silene latifolia* (Zluvova et al. 2001), and even in the different developmental phases of *Pinus* (Fraga et al. 2002) and *Prunus* (Bitonti et al. 2002). The earlier study with *Arabidopsis* has reported that young seedlings have lower DNA methylation levels than mature leaves (Finnegan et al. 1998). It has also been well evidenced that the change in DNA methylation do occur among the plants derived from in vitro tissue culture (Chen et al. 2009; Park et al. 2009) and somatic embryogenesis (Chakrabarty et al. 2003).

The molecular genetics for green seaweeds is still in their inception, and these organisms are useful source for investigating the molecular mechanisms underpinning the developmental processes. In the present study, genome-wide distribution and pattern of DNA methylation sites was studied for the first time in seaweed to investigate the epigenetic variations arising from protoplast-derived morphotypes. Further, the regenerated thallus types were shown to have interesting growth properties making their suitability for germplasm storage and propagation applications.

## Materials and Methods

### Collection of Seaweed Sample and Protoplasts Isolation

Thalli of *Ulva reticulata* Forsskål C were collected from Okha (22°27.04′ N; 69°03.58′ E), Gujarat, along the west coast of India and brought to the laboratory under cool conditions. The seawater temperature at collection site was 21°C. Selected thalli were thoroughly rinsed with autoclaved seawater to remove dirt and epiphytes. The unialgal culture of this alga was established by growing it in sterile enriched seawater media (Provasoli 1968) with GeO_2_ (10 mg L^−1^) for a week under white fluorescent lamps of irradiance intensity about 15 μmol photon m^−2^ s^−1^ with a 12:12 h light/dark photoperiod. During this period, the culture media were changed every 2 days. Thereafter, the algal thalli were made axenic and protoplasts isolation was carried out following the protocol described by Reddy et al. (2006) for green seaweeds.

### Protoplasts Culture

For protoplasts culture, a density of 1.0 × 10^5^ cells were dispensed into 10 ml of enriched seawater medium and incubated at a temperature gradient of 20 ± 1°C, 25 ± 1°C, 30 ± 1°C, and 35 ± 1°C under white fluorescent lamps of irradiance intensity about 15 μmol photon m^−2^ s^−1^ with a 12:12-h light/dark photoperiod in a plant growth chamber (Eyela, Japan).

### Cell Wall Regeneration

The regeneration of cell wall around fragile cell membrane of protoplasts was monitored after staining the protoplasts with 0.01% calcofluor white MR2 (Fluorescent brightener 28, Sigma Aldrich, USA). The stained cells were observed for blue fluorescence under epifluorescence microscope (Olympus model IX 70, Japan) equipped with fluorescent burner (BH2RFLT3, Olympus, Japan).

### Protoplasts Regeneration

The regeneration and differentiation pattern of protoplasts was regularly monitored under the inverted microscope (IX 70, Olympus, Japan). The regeneration rate of different morphotypes under the influence of temperature was determined by counting the differentiating cells in ten random microscopic fields in each plate. The number of sporulation events per thalli pattern was also recorded under all the conditions investigated.

### Daily Growth Rate (DGR %)

The length, breadth, or diameter according to the differentiating pattern of each morphotype was measured from the first cell division till the visible thalli were observed. The DGR (%) was measured for each developmental pattern on every third day after the first division. The DGR (%) was calculated according to the equation DGR (%) = [(*l*_*t*_/*l*_0_)^1/*t*^ − 1] × 100 where *l*_*t*_ and *l*_0_ are the length/diameter of filament/disk at time *t* and time 0, respectively. DGR (%) of the leafy thalli after regeneration was also calculated according to the equation DGR (%) = [(*W*_*t*_/*W*_0_)^1/*t*^ − 1] × 100 where *W*_*t*_ is the weight after time *t* and *W*_0_ is the initial weight at time *t* = 0.

### Statistical Analysis

Each experiment was repeated at least three times. All the data were reported as mean ± SD. For the comparison of the results under different conditions, analysis of variance was performed. Any statistical property *p* ≤ 0.05 was considered significant. The data of regeneration were correlated with the implementation of Pearson partition coefficient model, and its significance was considered valid at *p* ≤ 0.05.

### Methylation Sensitive Amplification Polymorphism Assay

Genomic DNA was extracted from both the protoplast-derived polymorphic thalli and normal wild-type thalli following the modified cetyltrimethylammonium bromide (CTAB) method. The thalli were gently blotted with tissue paper for removing surface water, ground to fine powder in liquid nitrogen, and mixed with preheated CTAB buffer (65°C) consisting of 2% CTAB (*w*/*v*), 1.4 M NaCl, 100 mM Tris–HCl (pH 8.0), 50 mM ethylene diamine tetra acetate (EDTA 2Na), 50 mM sodium sulfite, and 1% PVP and then incubated in water bath at 65°C for 1 h with occasional gentle mixing. The mixture was cooled to room temperature and then extracted twice with equal volume of chloroform/isoamyl alcohol (24:1), centrifuged at 12,000×*g* for 5 min, and upper aqueous layer was collected. The recovered aqueous layer was treated with RNase A (10 μg) for 30 min at 37°C. DNA was precipitated with isopropanol and pelleted by centrifugation at 12,000×*g*. The DNA pellet was washed with a mixture containing ethanol (76%) and sodium acetate (0.2 M). A second wash was followed with a mixture containing ethanol and 10 mM ammonium acetate, and the resulting pellet was air-dried. The final DNA pellet thus obtained was dissolved in Tris–EDTA (10 mM, pH 8.0). The purity of the extracted DNA was checked by OD_260_/OD_280_ ratio and agarose gel electrophoresis.

DNA methylation patterns of the developed morphotypes and wild-type plant were compared by methylation sensitive amplification polymorphism (MSAP) analysis according to the method described by Cervera et al. (2002) and Moghaddam et al. (2009). Briefly, genomic DNA of each thallus types were cleaved with restriction endonucleases *Eco*RI and either *Hpa*II or *Msp*I. The restricted fragments were ligated with *Eco*RI and *Hpa*II/*Msp*I adapters (Table 1) using T4 DNA ligase. Thereafter, primary PCR amplification was carried out using primers complementary to the *Eco*RI and *Hpa*II/*Msp*I adapters with one additional selective nucleotide at the 3′ end (Table 2) where ligated DNA fragments served as templates. PCR products thus obtained were diluted and used as templates for secondary selective amplification with combinations of primers complementary to the *Eco*RI and one *Hpa*II/*Msp*I adapters, but this time with two or three selective nucleotides, respectively, at the 3′ end (Table 3). The electrophoregram of the final PCR products was mapped on 2% agarose gel.

**Table 1:** Sequences of adapters in methylation sensitive amplified polymorphism assay

Adapter name	Sequence
*Eco*RI-adapterI	5′CTCGTAGACTGCGTACC 3′
*Eco*RI-adapterII	5′ AATTGGTACGCAGTC 3′
*Hpa*II/*MspI*-adapterI	5′ GACGATGAGTCTCGAT 3′
*Hpa*II/*Msp*I-adapterII	5′ CGATCGAGACTCAT 3′

**Table 2:** Pre-amplification primers complementary to the *Eco*RI and *Hpa*II/*Msp*I adapters with one additional selective nucleotide at the 3′ end

Adapter name	Sequence
*Eco*RI	5′ GACTGCGTACCAATTCA 3′
*Hpa*II/*Msp*I	5′ ATGAGTCTCGATCGGA 3′

**Table 3:** Secondary selective primer combinations complementary to the *Eco*RI and *Hpa*II/*Msp*I adaptors with two or three selective nucleotides at the 3′ end

Primers	Sequence
*Hpa*II/*Msp*I-AAT	5′ ATGAGTCTCGATCGGAAT 3′
*Hpa*II/*Msp*I-ATC	5′ ATGAGTCTCGATCGGATC 3′
*Hpa*II/*Msp*I-ACT	5′ ATGAGTCTCGATCGGACT 3′
*Eco*RI-AC	5′ GACTGCGTACCAATTCAC 3′
*Eco*RI-AA	5′ GACTGCGTACCAATTCAA 3′
*Eco*RI-AG	5′ GACTGCGTACCAATTCAG 3′
*Eco*RI-AT	5′ GACTGCGTACCAATTCAT 3′

### MSAP Data Analysis

MSAP data originated from the electrophoresis of PCR products were converted into a binary matrix of 1 and 0 based on presence or absence of band. An amplification pattern of the type 11 corresponded to samples displaying bands in both the *Msp*I and *Hpa*II lanes and considered for unmethylated site. Amplification patterns of the type 01 corresponded to samples showing an amplified band after restriction with *Msp*I but not after restriction with *Hpa*II (internal cytosine fully methylated site). Pattern 10 corresponded to samples displaying an amplified band after restriction with *Hpa*II but not after restriction with *Msp*I (external cytosine hemi-methylated site). Pattern 00 indicated no band amplified after restriction with either isoschizomer, revealing either full methylation at the locus in both cytosines or full methylation of the external cytosine. Two replicates of DNA extraction were performed from two different sets of cultured protoplasts. MSAP was also performed in duplicate to access the reproducibility and consistency of amplification profile.

For the statistical analysis of MSAP results, band-based strategy was adopted (Bonin et al. 2007). Methylation susceptible bands were scored in binary numbers as if the methylation state was assessed as the state of dominant markers. For this, band score = 1for the methylated state where bands were present in either of *Eco*RI–*Hpa*II or *Eco*RI–*Msp*I and band score = 0 for non-methylated state when bands were present in both the restriction cleavages. The epigenetic diversity was assessed as a measure of Shannon’s diversity index. Exact tests (*χ* ^2^) to yield exact probability of the observed differences in marker frequencies were calculated with Tools for Population Genetic Analysis (TPFGA) software using a MCMC approach (Miller 1997). The exact tests were performed with 1,000 dememorization steps, 20 batches, and 2,000 permutations per batch. *F* statistics were calculated with TPFGA and reported using the terminology of Weir where theta (*θ*) corresponded to *F*_ST_.

## Results

**Fig. 1:**
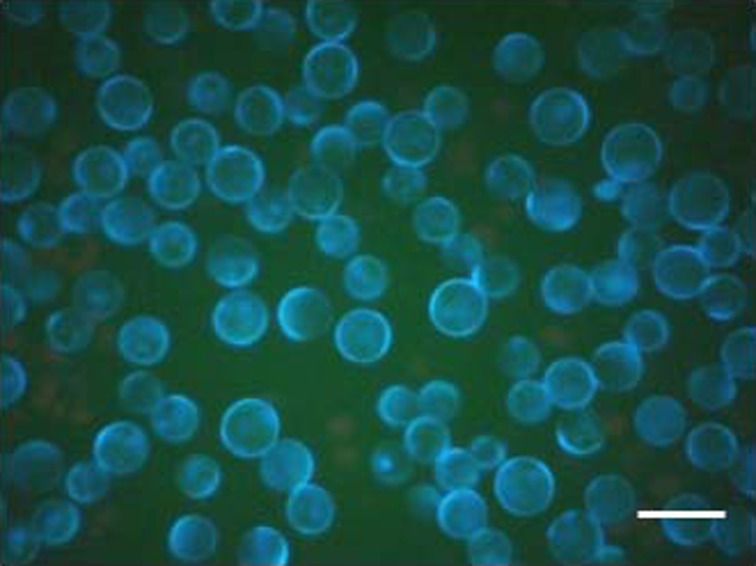
Deposition of cellulosic cell wall around fragile cell membrane of protoplasts as confirmed with calcofluor staining. *Scale bar* = 20 μm

**Fig. 2:**
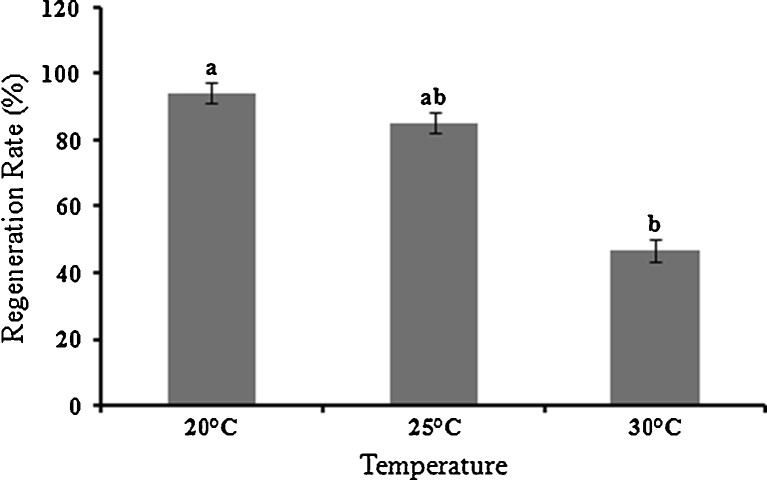
Regeneration rate of protoplasts of *U. reticulata* at different temperatures. Each datum is the mean of five replicates. *Vertical bars* indicate standard deviation. Significant differences are indicated with *alphabets on top of bars* (*p* ≤ 0.01)

**Fig. 3:**
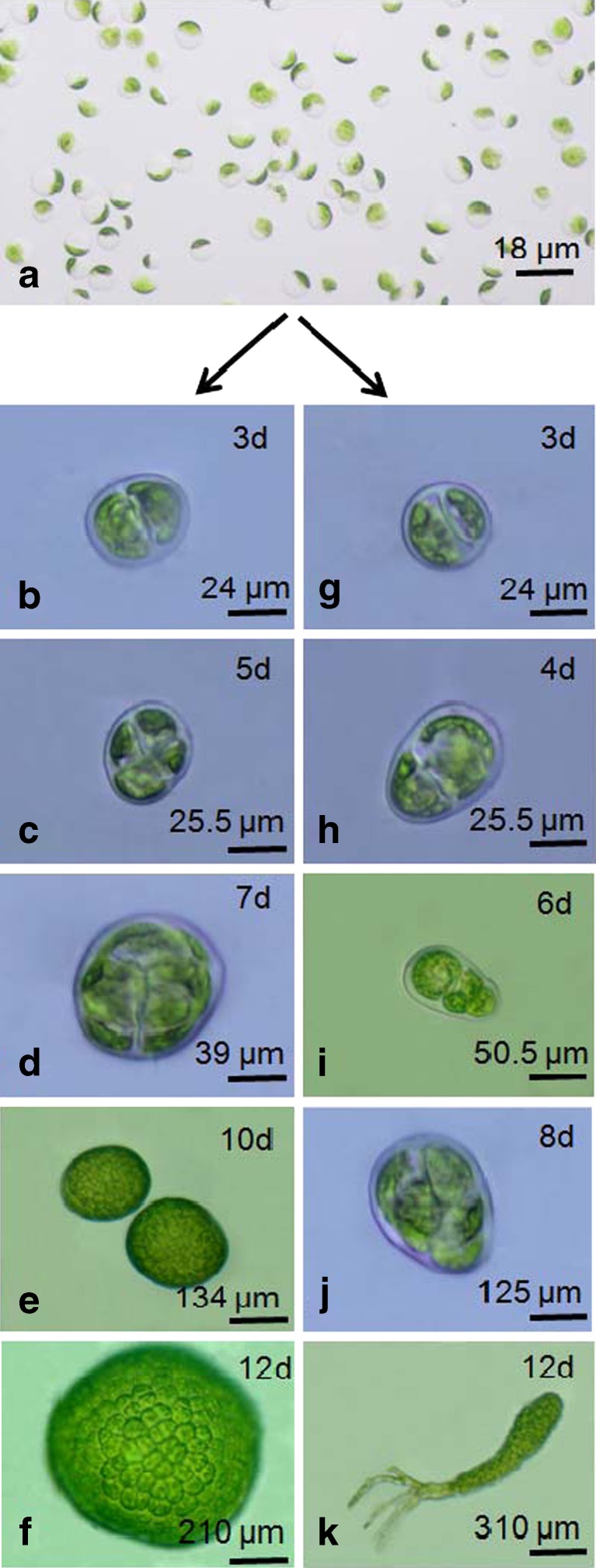
Differentiation of protoplasts (**a**) into variant disk-type thalli (**b**–**f**) and normal filamentous thalli (**g**–**k**)

The protoplasts yield of 1.4 ± 0.7 × 10^7^ cells g^−1^ fresh wt of size ranging 20–35 μm was obtained from *U. reticulata*. The spherical protoplasts started secreting microfibrillar cellulosic cell wall within 36–48 h in culture. The cell wall regeneration around the fragile cell membrane was confirmed by blue fluorescence of cultured cells after calcofluor staining (Fig. 1). There was a delay in the cell wall regeneration at temperature 30 ± 1°C while no regeneration of cell wall was observed at 35 ± 1°C. After development of cell wall, protoplasts started dividing in a homogenous manner. The protoplasts regeneration rate was optimum at 20 ± 1°C with 94.14 ± 3% followed by 85.29 ± 2.9% at 25 ± 1°C and significantly decreased to 46.8 ± 3.31% at 30 ± 1°C (*p* ≤ 0.01) (Fig. 2). The first division of the cell determined the differentiation and developmental patterns of viable protoplasts (Fig. 3). In the developmental pattern toward the regeneration of normal filamentous thalli, the first division was asymmetric and the cell divided transversely into unequal halves and developed heteropolarity. This is followed by second division perpendicular to first plane, resulted into three cell stage. Further periclinal division led to development of a dome shape structure with apical polarity. Initiation of rhizoids took place from the lower apex of the dome-shaped structure. The 8–10 days old germling had four to five rhizoids of size 90–125 μm, and the filament size ranged 125–200 μm (Fig. 3). In contrast, in another developmental pattern that resulted in regeneration of variant thalli, cell firstly divided across its diameter into equal halves forming two cell stage. This is followed with the second division perpendicular to the first axis resulting into four cell stage. Onward periclinal division resulted into eight cell stage where cells were arranged in the form of spherical disk of size 250–300 μm. Further transverse and parallel divisions enlarged the disk as hollow, unicellular, saccate-like structure. Also a third type of developmental pattern as sporeling phase was observed at temperature 30°C.

**Fig. 4:**
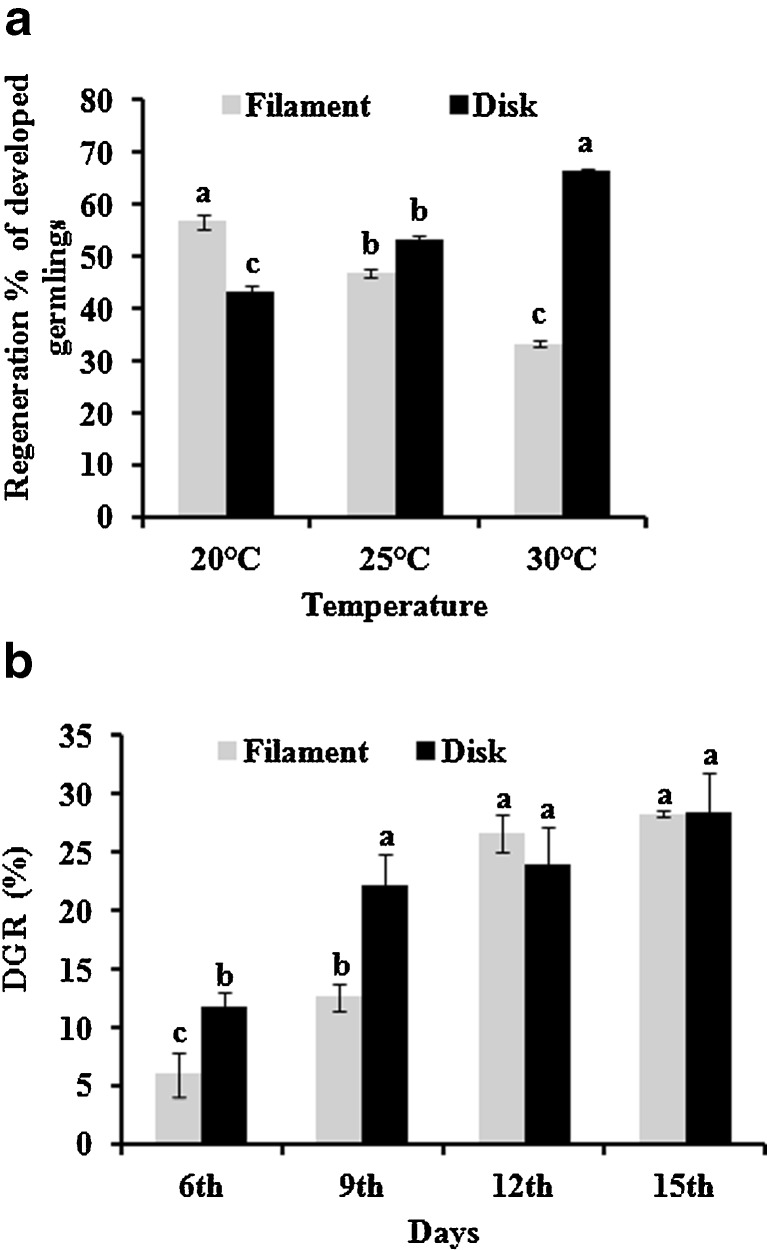
Regeneration rate of different morphotypes at different temperatures (**a**) and their daily growth rates (**b**). *Alphabets at top of bars* indicate significant differences among themselves (*p* ≤ 0.01)

The optimum regeneration rate of protoplasts was observed at low temperature (20 ± 1°C), but significant variations in the regeneration rate of the two morphotypes were observed. The regeneration rate for normal filamentous thalli was highest (56.84 ± 1.35%) at 20 ± 1°C and significantly decreased to 46.87 ± 0.81% at 25 ± 1°C and 33.43 ± 0.54% at 30 ± 1°C (*p* ≤ 0.01). On contrary, the other developmental behavior, i.e., disk-type thalli followed the reverse trend with highest regeneration of 66.54 ± 0.53% at 30 ± 1°C and significantly (*p* ≤ 0.01) decreased to 53.12 ± 0.81% and 43.16 ± 1.35% at 25 ± 1 and 20 ± 1°C, respectively (Fig. 4a).

The daily growth rate (DGR %) was determined for both the developing patterns at their respective optimum temperatures. The DGR (%) of normal filamentous pattern was found to significantly increase as 6.04 ± 1.87%, 12.66 ± 1.12%, and 28.31 ± 0.28% after 6, 9, and 15 days, respectively (*p* ≤ 0.01). Further, this germling state showed DGR (%) as high as 35.93 ± 1.16% in aeration flasks (Fig. 4b).

**Fig. 5:**
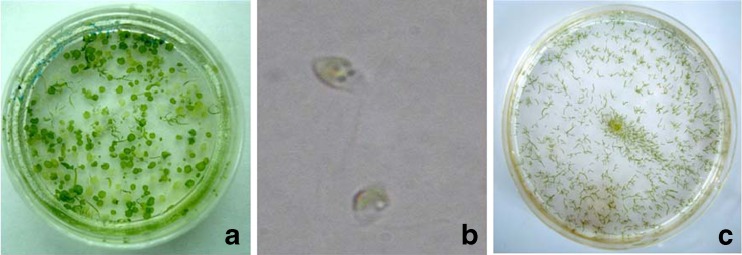
Development of normal filamentous thalli from disk-type germling. **a** Plate showing fully developed normal filamentous and disk-type morphotypes, **b** swarmers released from the disk-type morphotype, **c** regenerated normal filamentous thalli from the swarmers

The DGR (%) of hollow disk followed a significant increase (*p* ≤ 0.01) from 11.75 ± 1.3 at day 6 to 22.13 ± 2.77, 23.97 ± 3.31, and 28.4 ± 3.5 at 9, 12, and 15 days, respectively. The increment in the size of disk seized with time and attained a size maximum of 0.5–1.5 mm diameter after 20 days. The disk maintained itself in the same manner for more than 1 year at all the temperatures studied even without changing the culture medium for long durations. These disks when given sudden temperature shocks by shifting to higher temperature from their culture conditions released the swarmers (Fig. 5). The released swarmers when cultured at 20 ± 1°C developed into normal plantlets (Fig. 5). Also, these swarmers when cultured at different temperatures, as protoplasts cultured in this study, regenerated into normal filamentous thalli. However, an increase in incubation temperature led to increase in growth of basal rhizoids compared to the growth of apical filaments (Supplementary Fig. 1).

The Pearson partition coefficient model revealed negative correlation between the regeneration rate of the two thallus types under the influence of different temperatures and were found to be significant at *p* ≤ 0.01 with value of *r* = 0.9999. The DGR (%) of both the thallus types was in positive correlation (*r* = 0.89) at *p* ≤ 0.05.

The epigenetic variations among the protoplasts-derived germlings were assessed by MSAP assay. The MSAP assay is an AFLP-based technique that makes use of a pair of isoschizomers (*Hpa*II and *Msp*I) and their adaptors. This technique therefore has a limitation of not assessing specific genomic loci. The isoschizomers employed recognize the same tetranucleotide CCGG with differential sensitivity to methylation at inner or outer cytosine. Therefore, differential cytosine methylation markers can advantageously be examined without prior genomic knowledge of the sample. Of the 12 primer combinations employed for MSAP analysis, eight were successful in amplification and generated a total of 148 markers in both the developed morphotypes (Supplementary Fig. 2). A polymorphism survey was carried out within one morphotype (to assess occurrence of methylation) and among the morphotypes (to reveal differential methylation). Band arrangements were organized into categories depending upon the differential cleavage patterns obtained describing the possible methylation status (Table 4). Methylation status of the protoplast-derived morphotypes was also compared with wild-type plant to evaluate the effect of culture conditions. The results revealed a frequency of 32.43% as unmethylated DNA in normal filamentous thalli, 22.97% as fully methylated, 24.32% as hemimethylated, and 20.27% as methylation of internal cytosine at both the strands. The corresponding methylation status in disk-type thalli was 27.02%, 25.67%, 32.43%, and 14.86%, respectively. The genome-wide analysis showed that 53% of the methylation sites were polymorphic among the two morphotypes. The results showed higher frequency of hemimethylation and full methylation sites in disk type while complete methylation of internal cytosine was estimated higher in normal filamentous thalli. Methylation level in both the developed germlings is illustrated in Table 5. The MSAP results revealed no significant differences in the methylation status among the normal wild-type plant material and the protoplast-derived filamentous thalli. The estimated methylation frequencies were 25.80% (hemimethylation), 20.4% (methylation of internal cytosine), and 61.25% (total methylation). However, subtle difference in frequency of unmethylated sites (38.7%) was found in the wild plant compared to the protoplast-derived normal filamentous thalli (32.43%).

**Table 4:** Type and frequency of occurrence of different banding pattern and the methylation status among the two germling types

Pattern F/disk type	Methylation status		Number of bands	Frequency (%)
	Filamentous thalli	Disk-type thalli		
11/11	u	u	38	25.67
10/10	h	h	24	16.21
01/00	i	f	12	8.10
11/00	u	f	10	6.75
00/10	f	h	10	6.75
00/01	f	i	8	5.40
10/00	h	f	8	5.40
01/01	i	i	8	5.40
01/10	i	h	8	5.40
00/11	f	u	6	4.05
11/10	u	h	6	4.05
10/01	h	i	4	2.70
01/11	i	u	4	2.70
11/01	u	i	2	1.35

*u* unmethylated, *h* hemimethylated, *i* methylation at inner cytosine, *f* fully methylated

**Table 5:** Methylation levels of the two germling types developed

Methylation type	Filamentous thalli	Disk-type thalli
Unmethylated	48	40
Hemimethylated	36	48
Internal cytosine methylation	30	22
Full methylation	34	38
Hemi-methylation ratio (%)	24.32	32.43
Full methylation ratio (%)	22.97	27.14
Methylation ratio (%)	67.56	72.97

hemimethylation ratio = no. of hemimethylated bands/total bands full methylation ratio = no. of fully methylated bands/total bands methylation ratio = no. of hemimethylated + internal cytosine methylated + fully methylated bands/total bands

The frequency of methylated and non-methylated polymorphic loci showed marked difference as observed with frequency distribution of Shannon’s diversity index (*I*). Mean *I* for methylation susceptible polymorphic loci among the two morphotypes was (0.112 ± 0.03) which was significantly higher compared to the corresponding value for non-methylated loci (0.04 ± 0.01). Results of the chi-square (*χ* ^2^) contingency test indicated significant heterogeneity of marker frequency across polymorphic loci among the two morphotypes developed. The *F*_ST_ value among the epigenetic differentiable parameters (methylated and non-methylated) was 0.03 which is significant at *p* ≤ 0.01 after permutation tests with 1,000 repetitions.

## Discussion

In this study, differentiation of protoplasts of *U. reticulata* into different morphotypes under the same in vitro culture condition was correlated with the epigenetic variations arising from polymorphic DNA methylation pattern and distribution. The differentiation of protoplasts into various morphotypes has been reported for a wide range of seaweeds (Reddy et al. 1989; Huang et al. 1996; Chen 1998; Krishnakumar et al. 1999; Chen and Shih 2000; Rusig and Cosson 2001; Dipakkore et al. 2005). This is the first attempt made to understand the developmental polymorphism of seaweed protoplasts from the genome perspective. The higher methylation ratio in disk-type thalli compared to the normal filamentous thalli (Table 5) together with high degree of methylation polymorphism (53%) among the two thalli types was correlated with the differentiation inability of disk-type thalli. The results corroborate well with the studies on higher plants where higher methylation frequency influenced the differentiation ability of tissues cultured in vitro. Li et al. (2008) illustrated the differences in DNA methylation between undifferentiated suspension-cultured cells and young light-grown shoots of *Oryza sativa*. Higher methylation frequency of 10% was reported for regenerated juvenile leaf compared to the methylation frequency of 8% in mature leaf in potato explants cultured on heterotrophic and normal autotrophic media, respectively (Joyce and Cassells 2002). Also, *Acacia mangium* micropropagated in vitro resulted in different leaf morphologies, where higher methylation frequency of 22.4% was reported in juvenile leaf compared to mature leaf with 20.7% (Baurens et al. 2004). Chen et al. (2009) reported higher frequency of methylation (26.7%) in continuously developing protocorm-like bodies (cPLBs) compared to spontaneously differentiating PLBs (sdPLBs) (24.1%) from *Cymbidium hybridium*. Further, the frequency of full methylation was higher in sdPLBs over the cPLBs and methylation polymorphism among them was reported to be 80.1%. Also, higher methylation frequency (16.99%) was reported in the non-embryogenically developed callus of *Eleuterococcus senticosus* compared to the callus induced embryogenically (11.2%) (Chakrabarty et al. 2003). Fraga et al. (2002) reported an increase in the degree of reinvigoration due to a gradual decrease in the percentage of methylated cytosine, and this change in methylation could be of use to estimate morphogenic ability of the tissue. The DNA methylation is primarily controlled by the expression of family of DNA methyltransferase enzymes; however, NCBI GenBank search revealed no submission for such enzyme family from seaweeds. The genus *Ulva* has gained considerable attention as a model system for studying algal development due to its small genome size and simple thallus organization consisting of distromatic leafy blade with rhizoidal cells at base which fastens it up with substratum. The earlier studies were restricted to classical methods of plant physiology and microscopic approaches to understand the regulation of development in *Ulva* (Wichard and Oertel 2010). Therefore, the picture of seaweed epigenome analyzed in this study reflects the marked expression of epigenetic regulators and opens up new avenues for epigenetic engineering in seaweeds.

DNA methylation has been considered to control the genetic expression of specific tissue type, their age (Demeulemeester et al. 1999), and morphogenic ability (Zhang et al. 2006). Also, the somaclonal variations arising during micropropagation and somatic embryogenesis are known to exert changes in DNA methylation (Kovarik et al. 1997; Kaeppler and Phillips 1993). The stress conditions experienced by plantlets during micropropagation results in instability of plant genome and disturb the normal developmental controls. Differential DNA methylation mainly in 5′ upstream sequence of one or both the alleles affects the meristematic cell populations and developing parts and influence the expression of tissue specific genes (Fraga et al. 2002). The epigenetic modifications are also considered as adaptive process of cells under in vitro conditions (Miguel and Marum 2011). Epigenetic reprogramming in response to environmental stress during cell differentiation leads to adopt phenological and developmental plasticity as a regulatory mechanism. Phenotypic plasticity is considered to adjust the durations of various phenological phases in plants and also allows plants to avoid to the exposure of critical growth phases especially the reproductive phase to stress (Chinnusamy and Zhu 2009). The genus *Ulva* is characterized to have isomorphic diplohaplontic life cycle where no distinction between the life cycle stages is achieved. Even the gametophytic male and female thalli as well as sporophytic thalli have identical morphology and anatomy (Van den Hoek et al. 1995; Hiraoka and Yoshida 2010). However, in nature such hollow saccate like development do occur as an intermediate stage in developmental process in some genus like *Monostroma* (Tatewaki 1969), *Kornmannia* (Woolcott and King 1998), and *Gyralia* (Pellizzari et al. 2008). Nevertheless, the regenerated disk-type thallus in this study was an undifferentiated development as it was not having normal distromatic leafy blade and rhizoidal cells for attachment with substratum. Therefore, the disk type undifferentiated thalli is an adaptive developmental phase regenerated under the consequence of temperature stress exerted during in vitro differentiation culture conditions.

The present study not only investigated the genetic control for anomalous development in *U. reticulata* but also described the gradual developmental process of a single cell to specific thallus type. The study revealed the first division of protoplast as the determinant for the two ontogenic patterns resulted in *U. reticulata*. Interestingly, the disk-type phenotype developed was like dormant germlings similar to saccate like microthalli of *Ulva fasciata* described by Chen and Shih (2000). Earlier studies have shown that such anomalous developmental forms could be utilized as germplasm maintenance forms when cultured under specific environmental conditions for example germling cluster of *Ulva prolifera* were maintained in continuous flow of deep sea water and low temperature (Hiraoka and Oka 2008), micro-filaments of *Monostroma latissimum* were preserved for more than 3 years at low irradiance and low temperature (Chen 1998). Also the somatic cells of *Enteromorpha* were induced for developing the unorganized callus like structure by their culturing in higher density on solid media (Polne-Fuller and Gibor 1987). The disk-type germlings in this study were maintained for more than a year under laboratory conditions circumventing the need for specific incubation conditions. Further, disk-type thalli showed thermal stability as their regeneration rate was found to increase with temperature over the normal filamentous thalli with optimum regeneration rate at lower temperature (20°C). These results corroborate well with the findings of Chen (1998) where protoplasts of *M. latissimum* developed undifferentiated filaments at higher temperatures (30°C) and normal leafy thalli at low temperature (18–22°C).

The disk-type thalli in the present study were induced to sporulate simply by altering the incubation conditions to obtain the normal plantlets (Fig. 5). Wichard and Oertel (2010) reported that the sporulation in *Ulva* is controlled by intracellular secretion of “sporulation inhibitor factors” and secretion of these factors is governed by internal biological rhythm and by external stimuli such as temperature, pH, salinity, light intensity, and nutrients availability. The same mechanism could be attributed for synchronous release of swarmers from the disk-type thalli after sudden changes in temperature. Nevertheless, the zoospores released from wild population of *U. reticulata* never underwent such developmental dichotomy in vitro when cultured at various temperatures. However, higher growth of rhizoids was observed compared to apical filaments with increase in temperature (Supplementary Fig. 1). The thermal stability together with floating nature of the disk increases the population density with limit of space compared to attached thalli. Therefore, these disk-type thalli with their dormant physiology and control over sporulation substantiate their utility for in vitro germplasm maintenance and propagation.

Besides different environmental cues, the microflora associated with the seaweeds also regulates growth and morphogenetic ability of seaweeds (Singh et al. 2011; Marshall et al. 2006; Provasoli and Pintner 1980). Wichard and Oertel (2010) showed that seaweed associated bacteria secrete morphogenetic factors similar to plant hormones that control the thallus morphogenesis. Studies on higher plants have shown that the bacterial symbionts even induce changes in DNA methylation pattern (Grandbastien 1998). Therefore, DNA methylation is considered as a dynamic mechanism by means of which plasticity is induced by environmental (biotic and abiotic) and ontogenic signals (Kaeppler et al. 2000).

In conclusion, this study reveals that the frequency and distribution of DNA methylation as an important factor that perhaps regulates the morphogenesis of protoplast-derived regenerants. The protoplast-derived disk-type thalli substantiate their utility for in vitro germplasm maintenance and propagation.

## Electronic Supplementary Material

Below is the link to the electronic supplementary material.

Supplementary Fig. 1 Development of swarmers released from wild plant of *U. reticulata* at different temperatures (**A**) 20°C, (**B**) 25°C, and (**C**) 30°C (DOC 341 kb)

Supplementary Fig. 2 Methylation sensitive amplification polymorphism (MSAP) bands pattern in protoplast-derived morphotypes. *First lane in the gel picture 1* and *last lane in gel picture 2* refer to molecular weight marker (1.5 kb). The pair of lane after marker represents the methylation pattern in normal filamentous thalli alternatively followed by pair of lanes for disk-type thalli. *M* and *H* refer to digestion with *Eco*R1 + *Msp*I and *Eco*R1 + *Hpa*II respectively (DOC 337 kb)
