# Supplementary material for: Detection of Epigenetic Variations in the Protoplast-Derived Germlings of Ulva reticulata Using Methylation Sensitive Amplification Polymorphism (MSAP)
Source: Mar Biotechnol (NY). 2012 Feb 10;14(6):692–700. doi: 10.1007/s10126-012-9434-7 (PMC3494870; doi:10.1007/s10126-012-9434-7)
Supplement: Supplementary file 1 — Development of swarmers released from wild plant of U. reticulata at different temperatures (A) 20°C, (B) 25°C, and (C) 30°C (DOC 341 kb) [file 10126_2012_9434_MOESM1_ESM.doc]

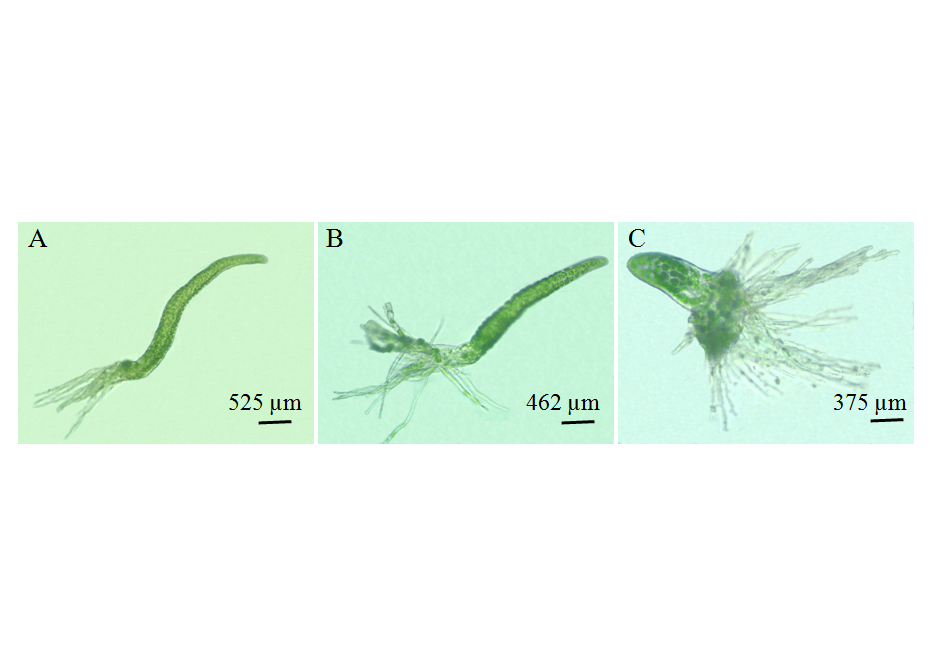


**Supplementary Fig. 1** Development of swarmers released from wild plant of *U*. *reticulata* at different temperatures (A) 20 °C, (B) 25 °C and (C) 30 °C
