# Supplementary material for: Detection of Epigenetic Variations in the Protoplast-Derived Germlings of Ulva reticulata Using Methylation Sensitive Amplification Polymorphism (MSAP)
Source: Mar Biotechnol (NY). 2012 Feb 10;14(6):692–700. doi: 10.1007/s10126-012-9434-7 (PMC3494870; doi:10.1007/s10126-012-9434-7)
Supplement: Supplementary file 2 — Methylation sensitive amplification polymorphism (MSAP) bands pattern in protoplast-derived morphotypes. First lane in the gel picture 1 and last lane in gel picture 2 refer to molecular weight marker (1.5 kb). The pair of lane after marker represents the methylation pattern in normal filamentous thalli alternatively followed by pair of lanes for disk-type thalli. M and H refer to digestion with EcoR1 + MspI and EcoR1 + HpaII respectively (DOC 337 kb) [file 10126_2012_9434_MOESM2_ESM.doc]

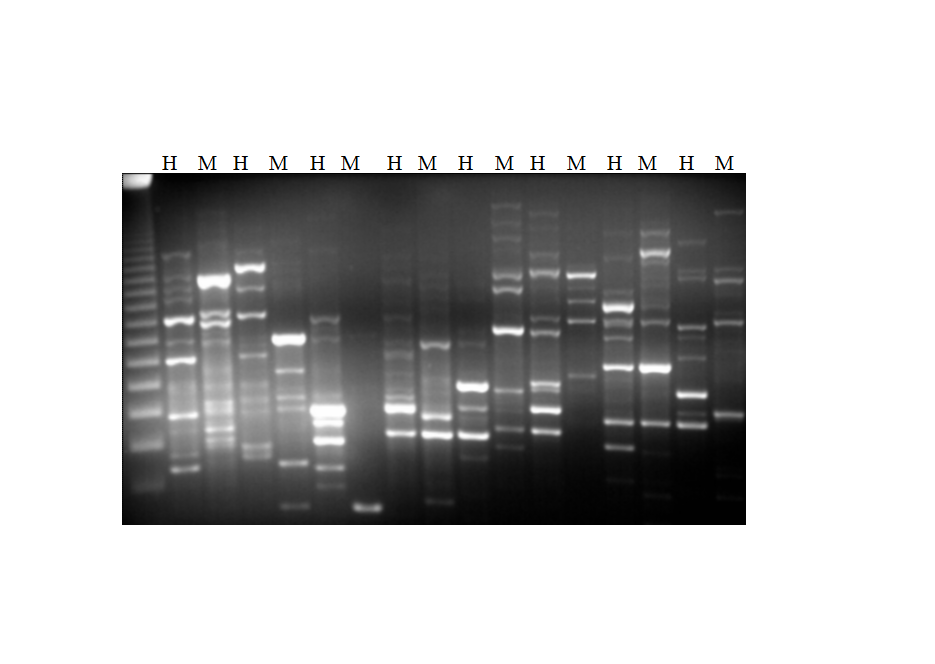

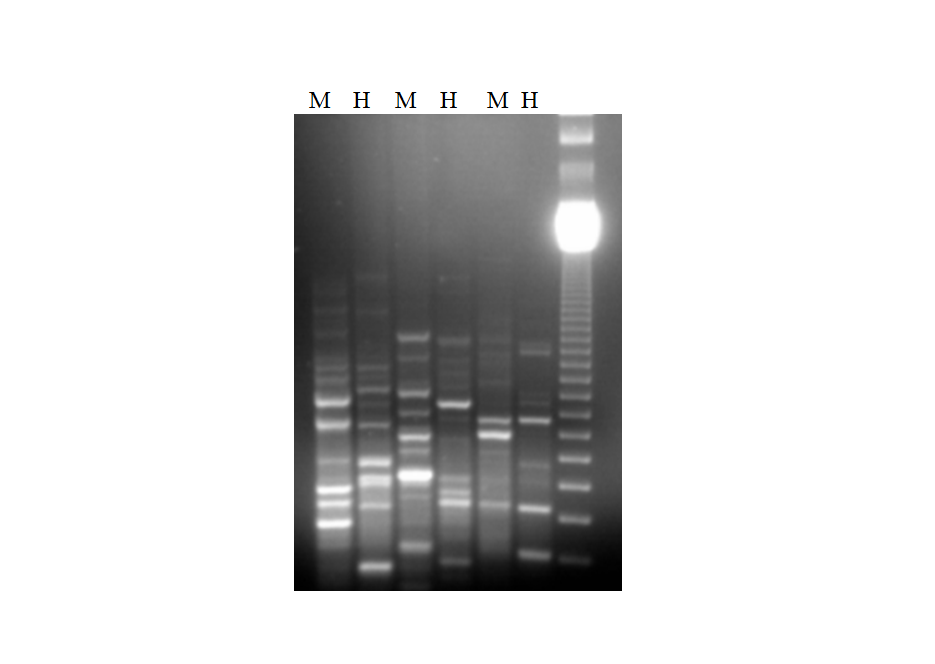


**Supplementary Fig. 2** Methylation sensitive amplification polymorphism (MSAP) bands pattern in protoplast-derived morphotypes. First lane in the gel picture 1 and last lane in gel picture 2 refer to molecular weight marker (1.5 Kb). The pair of lane after marker represents the methylation pattern in normal filamentous thalli alternatively followed by pair of lanes for disc type thalli. M and H refer to digestion with *Eco*R1 + *Msp*I and *Eco*R1 + *Hpa*II respectively
